# Substitutes for Mercurials in the Treatment of Syphilis

**Published:** 1852-04

**Authors:** 


*Substitutes for Mercurials in the Treatment of Syphilis.*—M. Robin read to the Academy of Sciences of Paris, a note on this subject, followed by a recital of the researches of M. Vicenti, also on the same question.

In a previous communication, M. Robin had enunciated the idea that mercurials do not exert any particular mode of action upon syphilitic disease, except in combining with the virus, and converting it into a new and inert compound. Many other substances, M. Robin had stated, possessed the same powers—*e. g.* preparations of arsenic, gold, silver, iron, and antimony, and therefore might advantageously replace the mercurial medication.

With this view, M. Vicenti had, at the request of M. Robin, studied experimentally the action of bichromate of potash. The following is a summhry of the results:

1. That the bichromate of potash possesses most undoubtedly anti-syphilitic properties more active and energetic than mercurial preparations.

2. That in the three cases in which it was administered no ill effects followed. The nausea occasionally excited is readily allayed by opium.

3. That being soluble the bichromate is rapidly taken into the system.

4. That the bichromate of potass may advantageously replace mercurials in the treatment of syphilis.—*Medical News and Library.*

